# Plasmon-enhanced photoluminescence from TiO_2_ and TeO_2_ thin films doped by Eu^3+^ for optoelectronic applications

**DOI:** 10.3762/bjnano.12.94

**Published:** 2021-11-22

**Authors:** Marcin Łapiński, Jakub Czubek, Katarzyna Drozdowska, Anna Synak, Wojciech Sadowski, Barbara Kościelska

**Affiliations:** 1 Institute of Nanotechnology and Materials Engineering, Gdansk University of Technology, Advanced Materials Center, Gabriela Narutowicza 11/12, 80-233 Gdańsk, Poland; 2 Faculty of Electronics, Telecommunications and Informatics, Digital Technologies Center, Gdansk University of Technology, Gabriela Narutowicza 11/12, 80-233 Gdańsk, Poland; 3 Faculty of Mathematics, Physics and Informatics, University of Gdańsk, Wita Stwosza 57, 80-308 Gdańsk, Poland

**Keywords:** gold nanostructures, luminescence, plasmon resonance

## Abstract

In this work we study the luminescence properties of europium-doped titanium dioxide and tellurium oxide thin films enhanced by gold plasmonic nanostructures. We propose a new type of plasmon structure with an ultrathin dielectric film between plasmonic platform and luminescent material. Plasmonic platforms were manufactured through thermal annealing of the gold thin film. Thermal dewetting of gold film results in spherical gold nanostructures with average dimensions of 50 nm. Both, luminescent TiO_2_:Eu and TeO_2_:Eu films were deposited by RF magnetron sputtering from mosaic targets. The morphology of the gold nanostructures was investigated by SEM and TEM, while the composition of oxides film was analyzed by XPS. Luminescence properties were studied on the basis of excitation and emission spectra. The experiments show that the additional dielectric layer enhances the luminescence intensity. Such structures could be potential candidates as phosphors in white LEDs.

## Introduction

The rapid development of optoelectronics leads to challenges in the search for new luminescence materials. Especially the fabrication of white LEDs requires more efficient phosphors. Potential new materials can be found through the computation of luminescent thin films and plasmonic platforms. Such a hybrid structure can be formed by thin oxide layers doped with rare-earth ions deposited on metal nanostructures [1–3]. Plasmonic resonance can be observed in metallic nanostructures, so-called plasmonic platforms, and thin films. Among different plasmonic materials, gold nanostructures exhibit resonance in the visible range and have been extensively studied as a material for light absorption and emission improvement [4–8]. Titanium dioxide seems to be one of the most popular and widely used oxide material as matrix for rare-earth ions [9–13]. Tellurium dioxide can be also considered as excellent in hosting rare-earth ions because of its low phonon energy (ca. 700–800 cm^−1^), which allows to minimize non-radiative losses [14–16]. Modification of oxide thin films can be implemented in many ways. The change of deposition parameters and working gasses, doping with various elements, or annealing at an elevated temperature are the most commonly used procedures [17–18]. It gives enormous possibilities for manufacturing thin films with novel properties and opens fields of new applications. Especially, doping of oxide materials by rare-earth ions positively influences the luminescent properties. Rare-earths ions represent a vast group of luminescent materials that exhibit light emission in the visible range [19–20]. Among them, europium (Eu) has been intensely studied for a few decades. Although Eu usually assumes a trivalent oxidation state (Eu^3+^), the divalent state (Eu^2+^) is also stable, but characterized by different luminescent properties. The Eu^3+^ emission spectrum consists of sharp lines, which are ascribed to ^5^D_0_→^7^F*_J_* (*J* = 0–6) transitions (intra-configurational 4f transitions), observed in the range of 570–840 nm [21–23]. As a red light emitter, Eu^3+^ may be employed in various optical devices.

In this work we compare two types of matrix for europium ions, namely titanium dioxide and tellurium dioxide. We show possibilities to enhance the luminescence by plasmon resonance. These nanostructures could find practical applications, for example, as phosphor material in LEDs.

## Experimental

**Figure 1:**
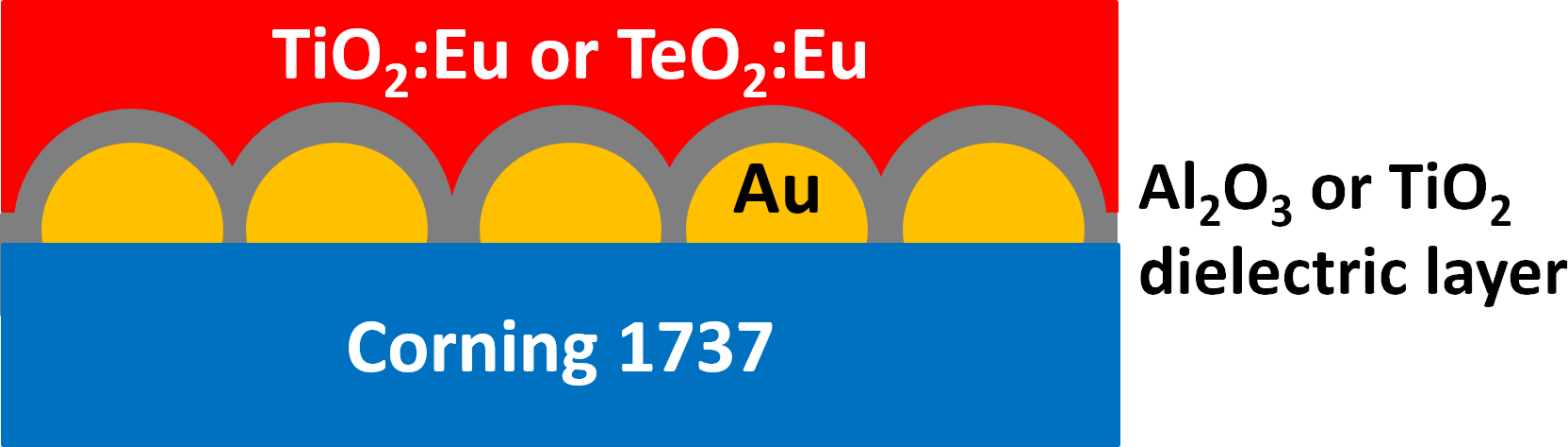
Schematic view of the prepared structures.

Corning 1737 glass was chosen as a substrate for film deposition. The substrates were gently cleaned with warm acetylacetone, then rinsed in ethanol and dried at 50 °C. Plasmonic nanostructures were prepared by thermal dewetting of gold thin films. Thin Au films with a thickness of 2.8 nm were deposited using a tabletop DC magnetron sputtering coater (EM SCD 500, Leica) in pure Ar plasma (argon, Air Products, 99.999%) at a pressure of 0.2 Pa. The round Au target with 99.99% purity was sputtered by 30 W of incident power. The rate of Au layer deposition was about 4 Å/s. Thickness was controlled by a built-in quartz crystal microbalance. As prepared films were subsequently put in a hot furnace for the formation of nanostructures. Samples were annealed at 550 °C for 15 min in air atmosphere. The formation of metallic nanostructures has been described in detail in our previous works [24–26]. On the prepared plasmonic platforms a dielectric buffer layer was deposited. We chose two kinds of layers. The first one, Al_2_O_3_, with different thicknesses in a range of 2 to 8 nm was deposited by atomic layer deposition (ALD) using a Beneq TFS 200 ALD system. This method provides precise control over the thickness with atomic accuracy. Trimethylaluminum (Sigma-Aldrich) and purified water were used as precursors. The deposition of the atomic layers was conducted at 200 °C. The second kind of dielectric layer was TiO_2_. It was prepared by radio frequency (RF) reactive magnetron sputtering using an Omicron Nanotechnology four targets sputter system. A Ti target (99.9%) was sputtered in an argon–oxygen atmosphere (Ar/O_2_ flow ratio: 5 sccm:30 sccm, both gasses from Air Products, 99.999%). The used RF power of 60 W resulted in the deposition with a ratio ca. 0.1 Å/s. Luminescent TiO_2_:Eu or TeO_2_:Eu films were deposited by RF magnetron sputtering. Metallic Ti–Eu (99.9%) or Te–Eu (99.9%) mosaic targets with a diameter of 50.8 mm were sputtered for about 50 min in argon–oxygen atmosphere (Ar/O_2_ flow ratio: 5 sccm:30 sccm) at a power of 40 W. This resulted in deposited films with a thickness of ca. 300 nm (deposition rate ca. 0.1 Å/s). The deposition process of TeO_2_:Eu films was described in our previous work [27]. The preparation of all TiO_2_, TeO_2_:Eu, and TiO_2_:Eu layers was conducted at 200 °C. The pressure in the chamber was approximately 0.2 Pa and the distance between target and substrate was approximately 10 cm. The sputtering system was equipped with a quartz crystal microbalance for the in situ measurements of film thickness. In order to obtain uniform heating of substrates and homogeneous films, a rotation of sample holder equal to 1 rpm was employed. All deposition parameters are collected in Table 1. A schematic view of the prepared samples is presented in Figure 1.

**Table 1:** Magnetron sputtering parameters.

Deposited material	Au	TiO_2_	TiO_2_:Eu	TeO_2_:Eu

target	Au	Ti	Te-Eu (mosaic)	Ti-Eu (mosaic)
power supply	DC	RF	RF	RF
power (W)	30	60	40	40
gas composition Ar/O_2_ (sccm)	100:0	5:30	5:30	5:30
working pressure (Pa)	0.2	<0.2	<0.2	<0.2
deposition rate (Å/s)	4	0.1	0.1	0.1

To analyze the morphology of the plasmonic platforms, a Zeiss CrossBeam 540 scanning electron microscope (SEM) operated at 2 kV was used. For microstructure analysis of the plasmonic structures, a TALOS F200X high-resolution transmission electron microscope (HRTEM) was used. The chemical composition of the luminescent layers was investigated by X-ray photoelectron spectroscopy (XPS). Measurement was performed using Omicron Nanotechnology equipment at room temperature and under ultrahigh vacuum conditions, at a pressure below 1.1 × 10^−6^ Pa. A Mg Kα X-ray source was operated at 15 kV and 300 W. XPS analysis were performed using CASA XPS software package with Shirley background subtraction and the least-square Gaussian–Lorentzian – GL(30) curve fitting algorithm. Calibration of obtained spectra to the binding energy of 285 eV for C 1s was conducted. Additionally, a built-in Ar ion gun was used to etch the surface of the films. To obtain depth profiles of the chemical composition, layers were etched for 2, 6, and 8 min. Optical transmittance spectra of all prepared structures were measured using an Evolution 220 UV–visible spectrophotometer in the range of 200–1000 nm. Luminescence excitation and emission spectra were recorded using a Scinco FluoroMate FS-2 fluorescence spectrometer. Excitation spectra were monitored at the wavelength of λ_em_ = 615 nm in a range of 350–460 nm, whereas emission spectra were collected in a range of 560–630 nm with an excitation wavelength of λ_exc_ = 394 nm.

## Results and Discussion

### Plasmonic platforms

**Figure 2:**
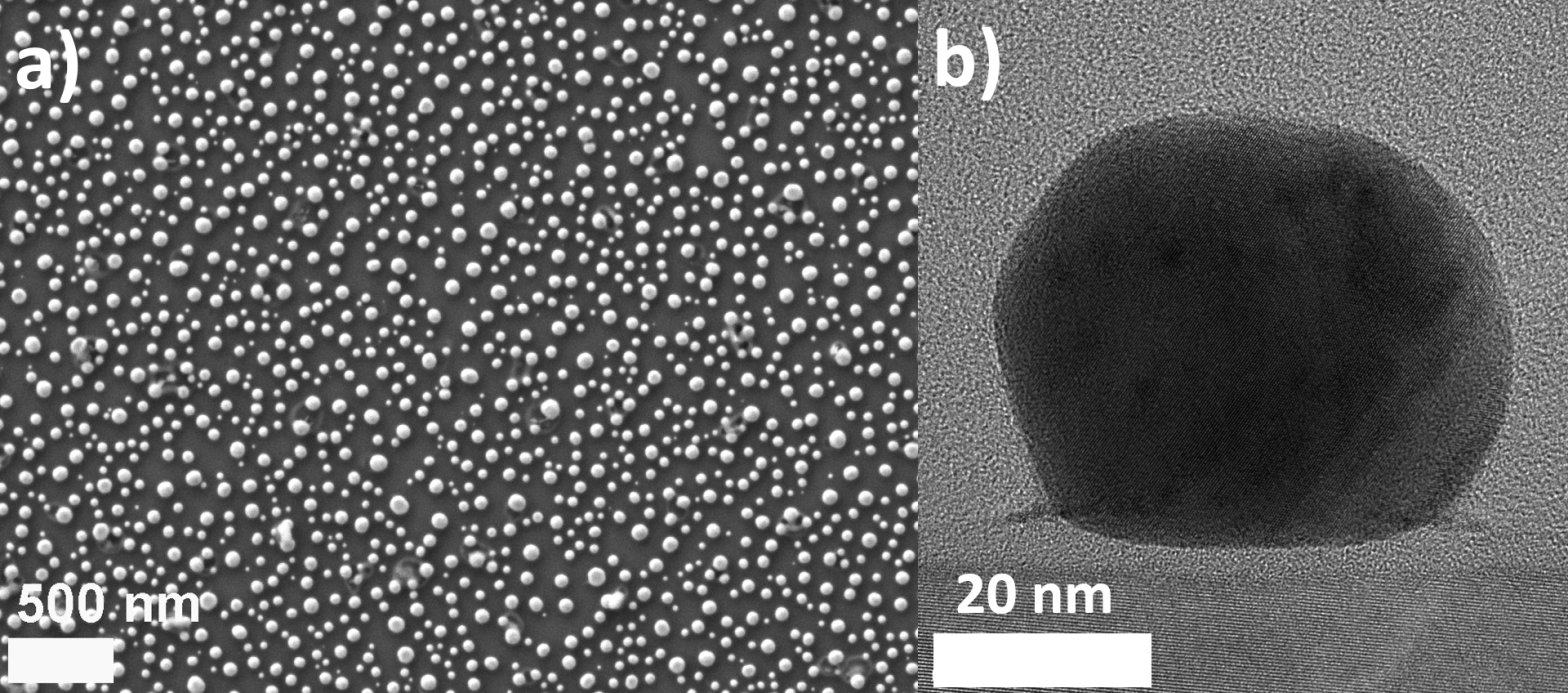
(a) SEM image of gold plasmonic platform, (b) HRTEM image of the cross section of a single gold nanoisland [25]. Figure 2a,b was reproduced from [25] (© 2019 M. Łapiński et al., published by Springer Nature, distributed under the terms of the Creative Commons Attribution 4.0 International License, https://creativecommons.org/licenses/by/4.0).

**Figure 3:**
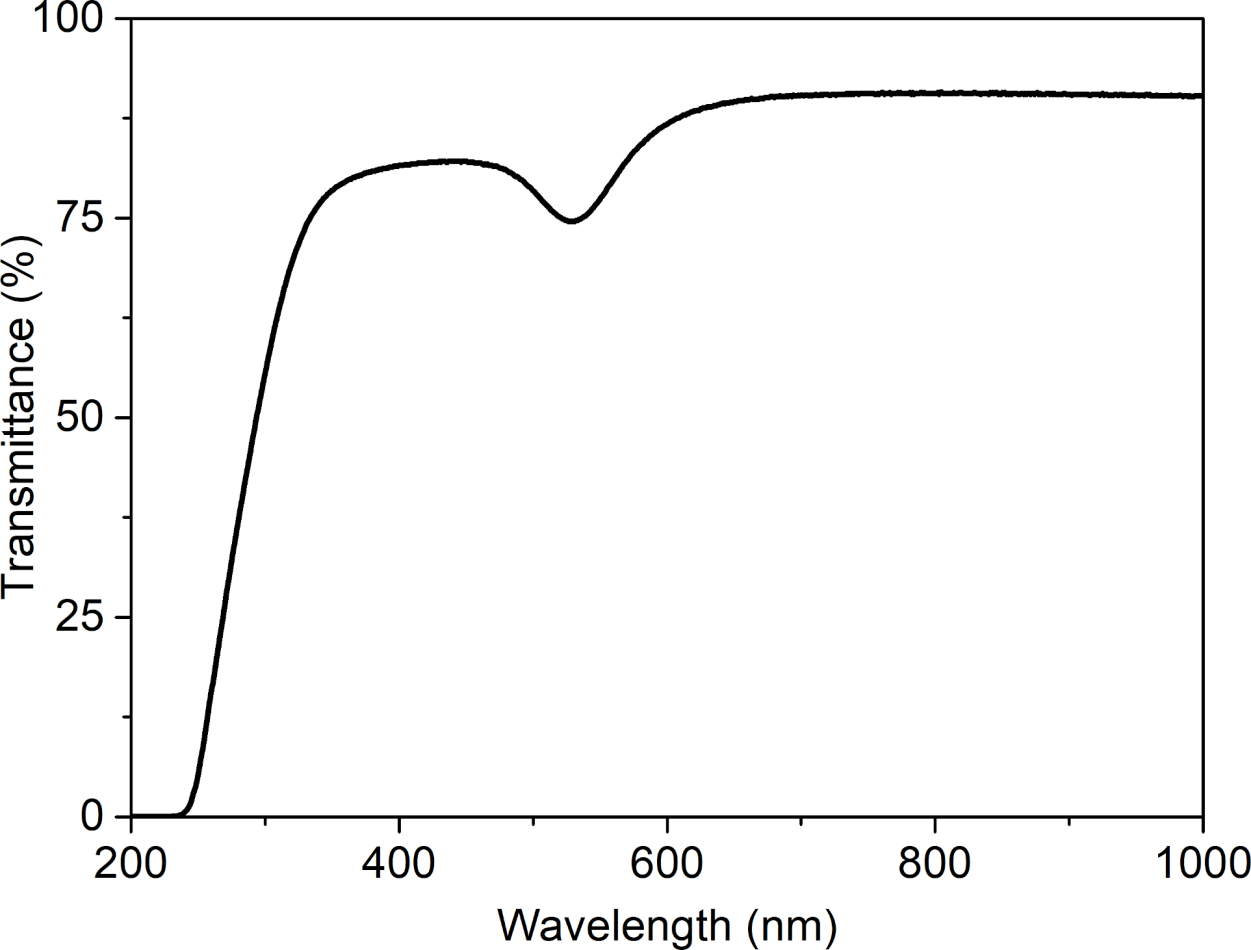
Transmittance spectrum of a gold plasmonic platform.

The quality of the gold plasmonic platforms was examined by SEM and TEM. The SEM image presented in Figure 2a shows a good uniformity of the prepared Au nanostructures. Nanoislands cover the whole substrate surface. Additionally, the HRTEM image of a cross section of a single nanoisland is shown in Figure 2b [25–26]. It can be seen, that the nanostructure with a diameter of ca. 50 nm is not perfectly spherical, but flattened on the substrate side. An exemplary transmittance spectrum recorded for the plasmonic platform is presented in Figure 3. A strong transmittance minimum, corresponding to plasmon resonance is observed at about 530 nm. The rapid decrease of transmittance at 350 nm is caused by absorption of glass substrate and can be noticed for other samples as well.

### TiO_2_:Eu-based structures

**Figure 4:**
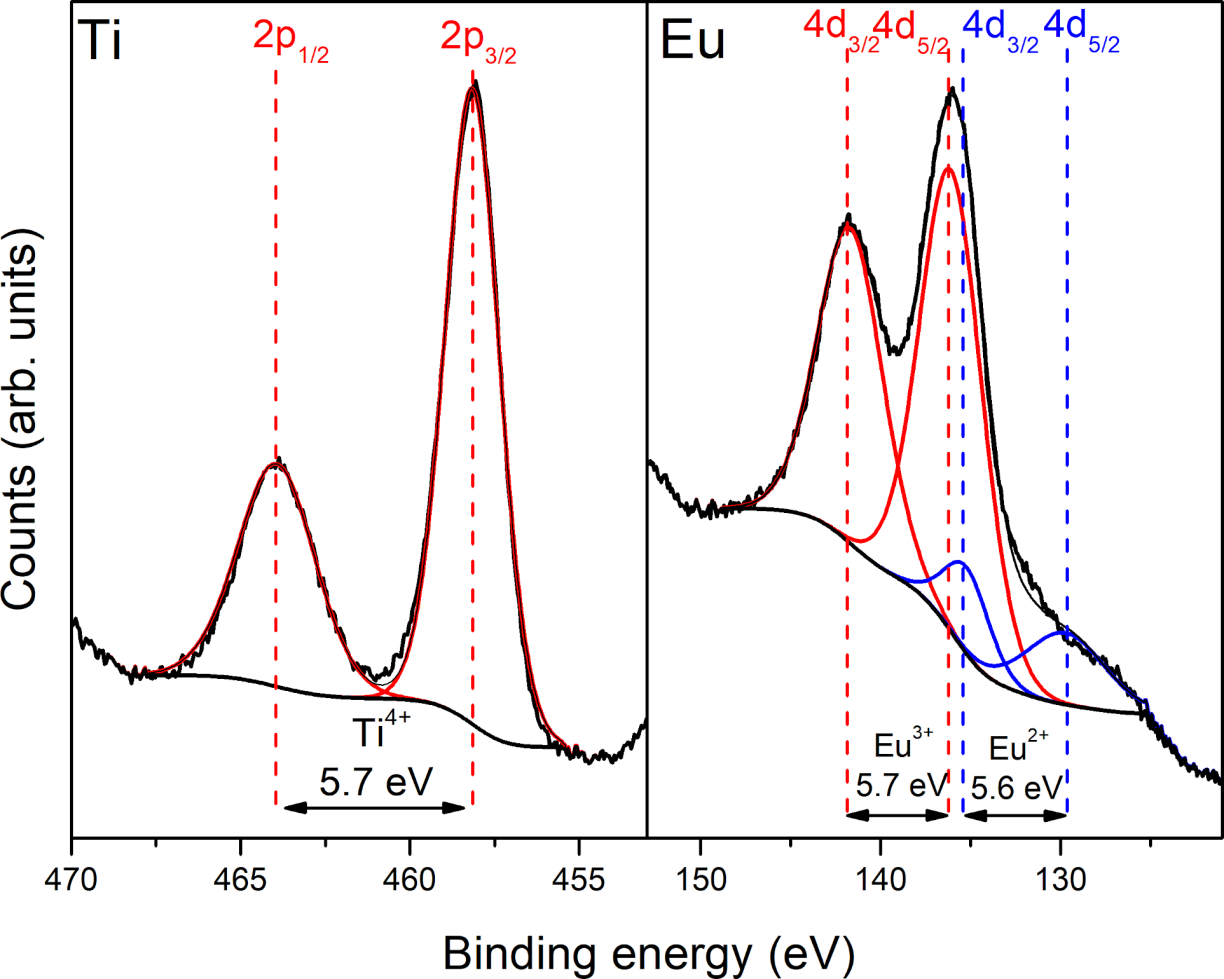
XPS spectra of Ti2p and Eu4d spectra of TiO_2_:Eu luminescent film.

The chemical composition of the luminescent titanium dioxide doped with europium was examined using XPS. The spectra of Ti 2p and Eu 4d core level electrons are presented in Figure 4. The experimental curve can be deconvoluted in two peaks at 458.2 eV and 463.9 eV, with a splitting energy of 5.7 eV, what is characteristic for Ti^4+^ [28–30]. Deconvolution of the Eu 4d spectrum is complicated. As it can be seen in Figure 4, the sample includes both Eu^3+^ and Eu^2+^ ions [31–32]. The Eu 4d doublet separation was considered as 5.7 eV for Eu^3+^ and 5.6 eV for Eu^2+^. The doublet that is assigned to Eu^3+^ ions dominates over the Eu^2+^ doublet. This led to the conclusion that the quantity of Eu^3+^ ions far exceeds that of Eu^2+^ ions. The calculated ratio Eu^3+^/Eu^2+^ was ca. 90%:10%. Additionally, on the basis of the survey spectrum the total percentage content of europium in the TiO_2_:Eu layer was calculated to be 5%.

**Figure 5:**
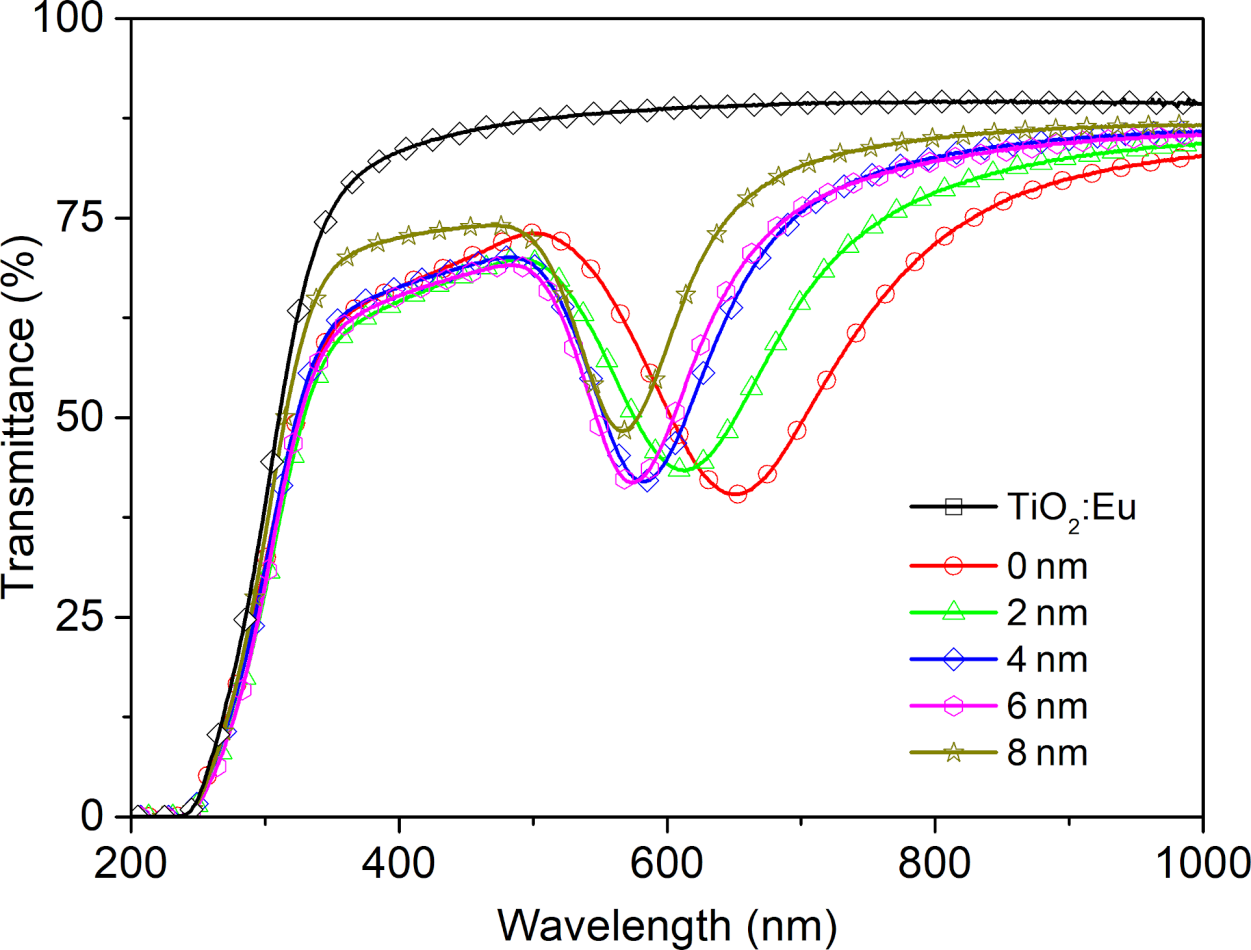
UV–vis spectra of a TiO_2_:Eu film and TiO_2_:Eu films deposited on the plasmonic platform without a dielectric layer (0 nm) and with a dielectric Al_2_O_3_ layer with different thickness, in a range of 2 to 8 nm.

**Figure 6:**
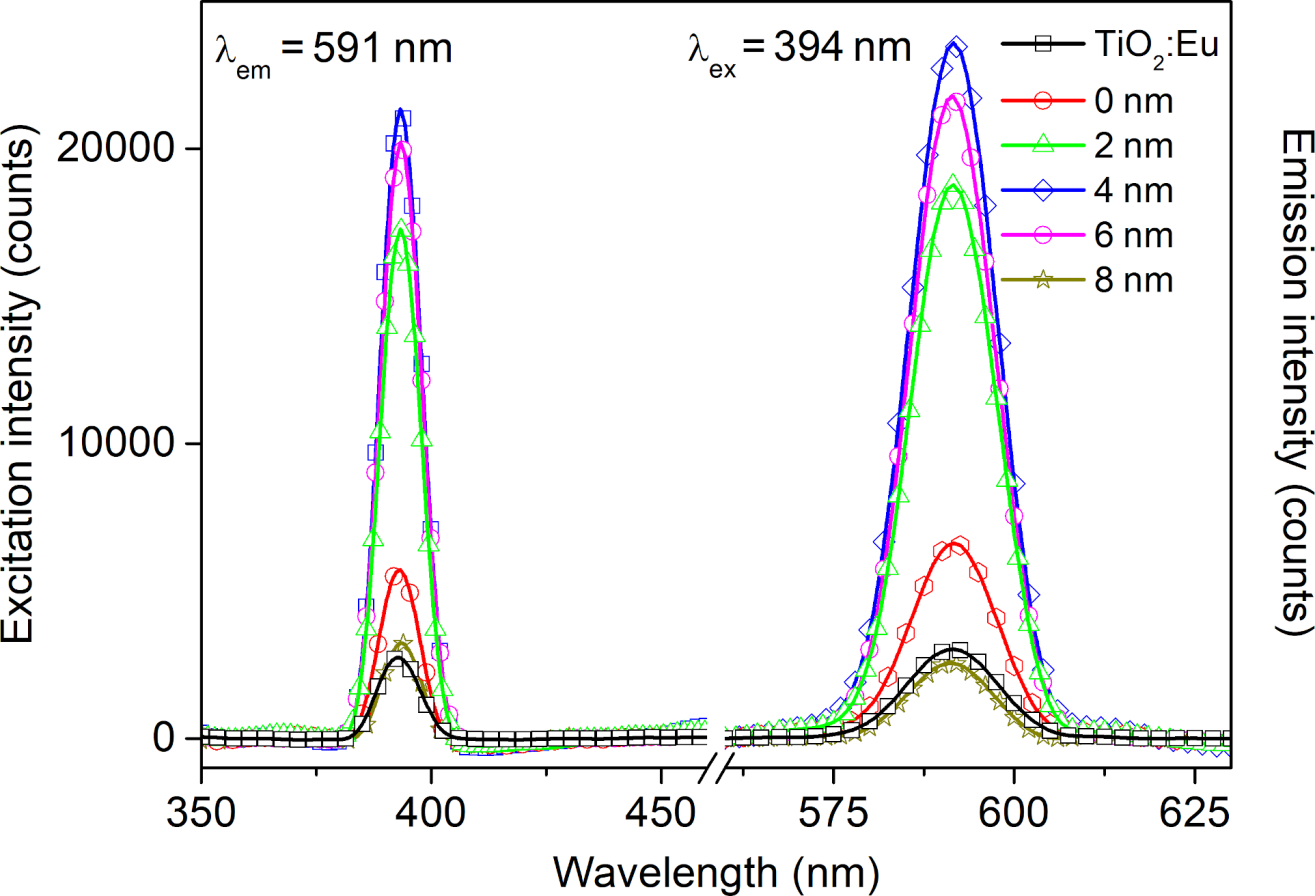
Excitation and emission spectra of a TiO_2_:Eu film and TiO_2_:Eu films deposited on the plasmonic platform without a dielectric layer (0 nm) and with a dielectric Al_2_O_3_ layer with different thickness, in a range of 2 to 8 nm.

Figure 5 shows the UV–vis transmittance spectra for all prepared TiO_2_:Eu structures. It can be seen that the TiO_2_:Eu thin film deposited directly on the glass substrate has a flat transmission characteristic with a transmittance of ca. 85%. For the luminescence layer deposited directly on a plasmonic platform, without dielectric film, a transmission minimum at 650 nm is visible. It corresponds to the surface plasmonic resonance effect and is strongly redshifted in comparison to plasmon platform characteristics (Figure 3). This phenomenon is directly related to Mie’s theory and can be caused by changes of the electric permittivity over the gold nanostructures [7–8,33]. A shift is also observed in structures with an additional ultrathin Al_2_O_3_ film. However, a blueshift occurs here due to the electrical properties of aluminum oxide. Additionally, it can be seen, that the position of the minimum of transmission as function of the Al_2_O_3_ film thickness. This may be explained by the different permittivity of the layers [34–36]. Emission and excitation spectra are shown in Figure 6. In the excitation spectrum in the range of 350–460 nm only one peak at 394 nm can be observed. The maximum at that peak corresponds to the ^7^F_0_→^5^L_6_ electric dipole transition for Eu^3+^ [22,37]. The peaks intensities are strongly enhanced when the luminescent layer is deposited on the plasmonic platform with a dielectric Al_2_O_3_ layer. The most intense peaks appear for films with 4 and 6 nm thickness. The emission spectra show only one significant peak at 591 nm. It corresponds to the ^5^D_0_→^7^F_1_ magnetic dipole transition. This type of transition is observed in centrosymmetric structures. This led to the conclusion that there might be some nanocrystal areas in the TiO_2_:Eu films. It is in line with our previous structural investigations. XRD measurements showed an amorphous or nanocrystalline structure of the oxide layers deposited by magnetron sputtering [31]. The intensity of luminescence is enhanced for structures with additional dielectric layer and is the highest for the TiO_2_:Eu layer deposited on plasmonic platforms with 4 or 6 nm of Al_2_O_3_. In contrast, a dielectric layer with a thickness of 8 nm completely neutralizes the impact of the plasmonic platform. The increase of luminescence for samples with plasmonic nanostructures can be explained by a local concentration of the electric field around the nanostructures. It could increase the rate of excitation [3]. The additional Al_2_O_3_ dielectric layer separates plasmonic gold nanostructures and TiO_2_:Eu luminescent film, which changes the conditions of electromagnetic interaction between plasmons and luminescent material.

### TeO_2_:Eu-based structures

**Figure 7:**
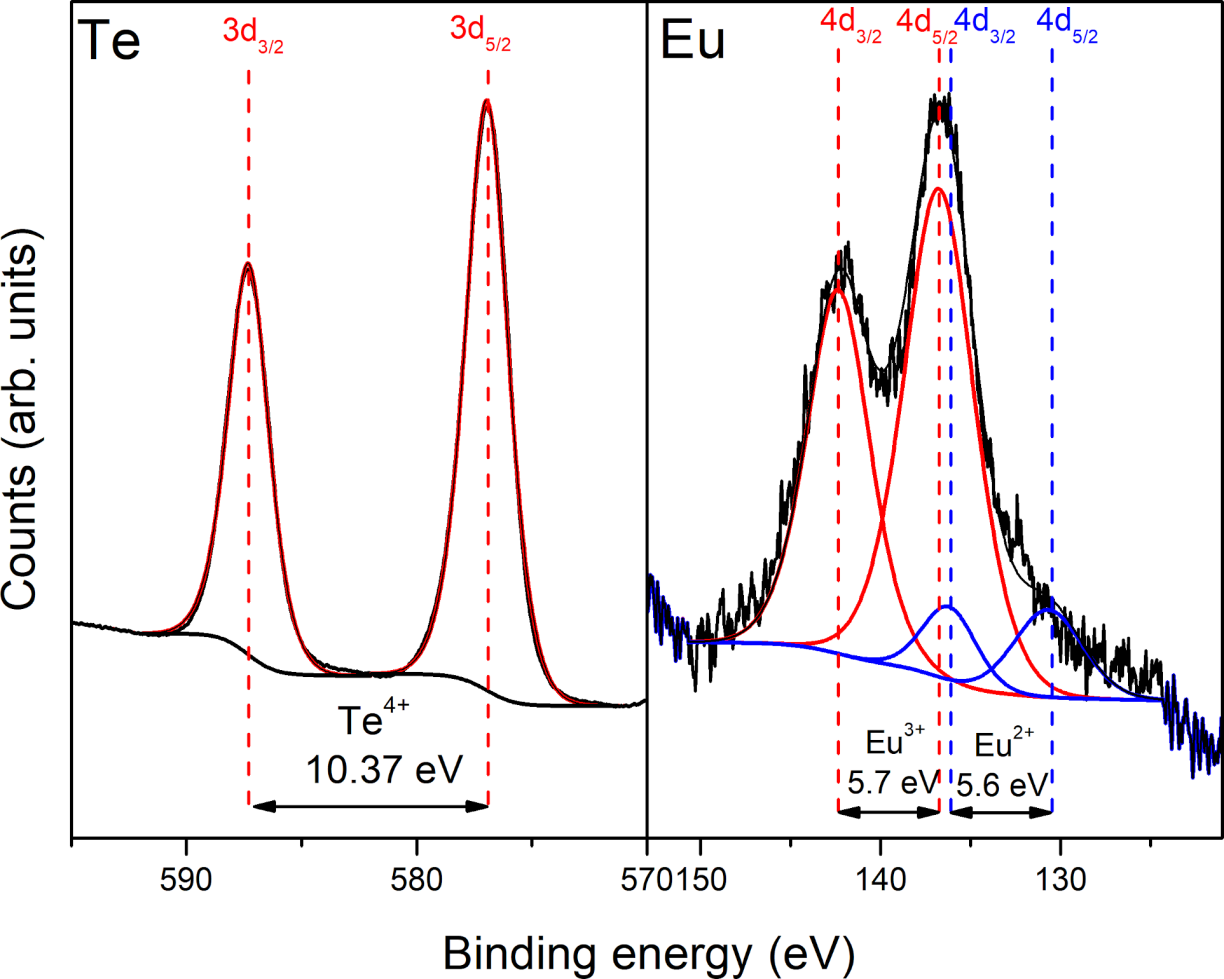
XPS spectra of Te 3d and Eu 4d regions of the TeO_2_:Eu luminescent layer.

**Figure 8:**
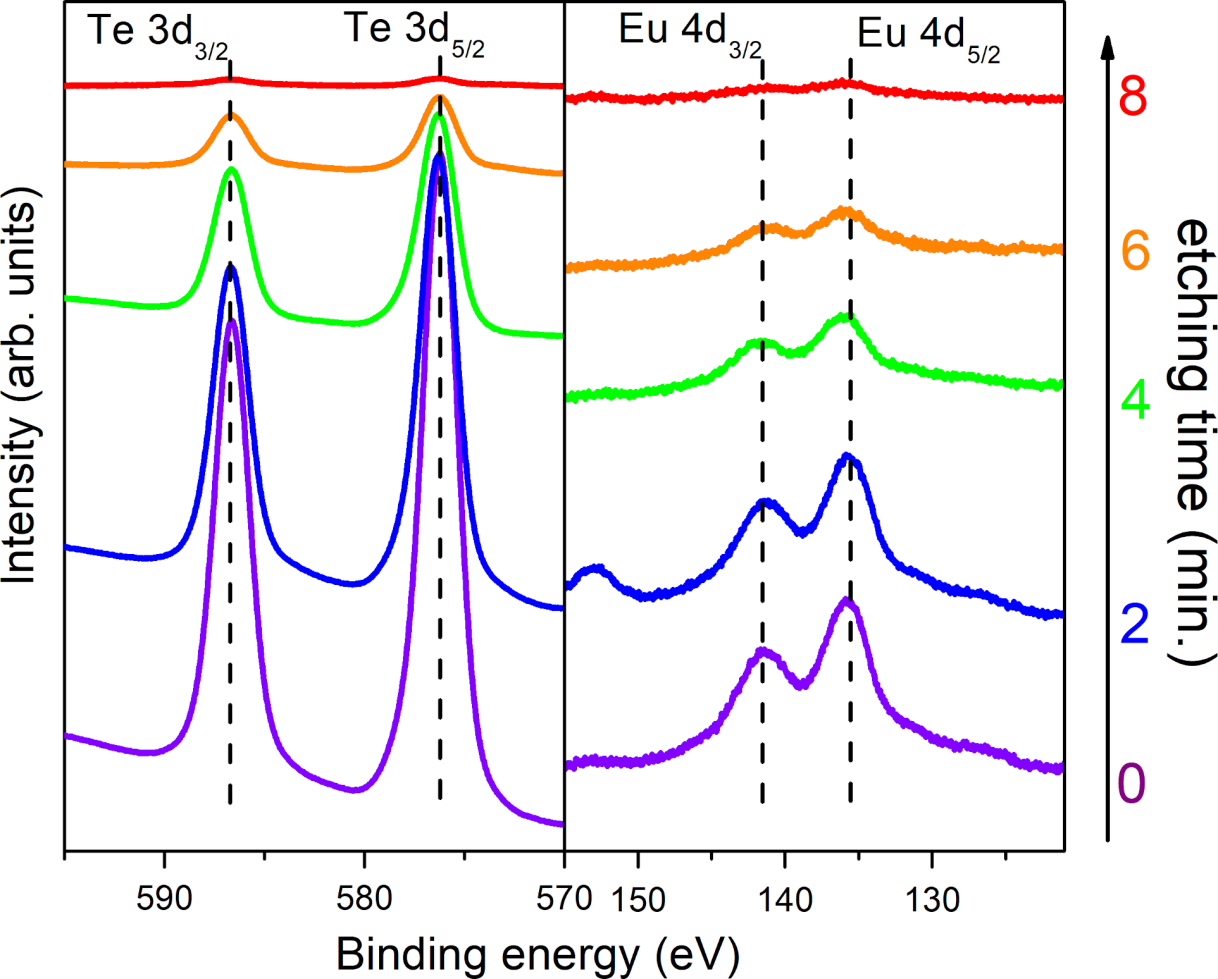
Depth profile of the chemical composition of TeO_2_:Eu thin film.

The results of the XPS analysis of TeO_2_:Eu luminescent layer are presented in Figure 7. Two peaks at 587.3 and 576.07 eV, with an energy separation of 10.37 eV, correspond to tellurium in the fourth valence state (Te^4+^) and are consistent with literature data [27,38]. The highly oxidative atmosphere during the sputtering process results in a lack of metallic tellurium in the deposited film. The Eu 4d spectrum can be deconvoluted into two doublets, which suggests the presence of europium in two valence states. Lines recorded at 142.36 and 136.76 eV correspond to Eu^3+^, while those observed at 136.40 and 130.90 eV were assigned to Eu^2+^. The energy separation in both doublets equals to 5.60 and 5.50 eV for Eu^3+^ and Eu^2+^, respectively, in accordance with literature [31–32]. The Eu^3+^ doublet is characterized by greater intensity and dominates over the Eu^2+^ lines. For quantitative analysis, integral intensities of europium doublets were compared. The Eu^3+^/Eu^2+^ ratio was ca. 90%:10%. Total amount of dopant in a sample was calculated to ca. 5%, on the basis of survey spectra. The amount of luminescent dopant in both titanium dioxide and tellurium dioxide matrixes should be sufficient to observe luminescence from the thin films. According to literature, percentage of luminescent ions in the host material should not exceed 10%. Otherwise, emission quenching may occur [39]. Additionally, a depth profile of concentration was measured. XPS results after etching of the TeO_2_:Eu film showed good chemical uniformity over the whole thickness of the layer, which is shown in Figure 8. After 8 min of etching the film was completely removed from the Corning glass substrate and no signals from Te 3d and Eu 4d lines were detected.

**Figure 9:**
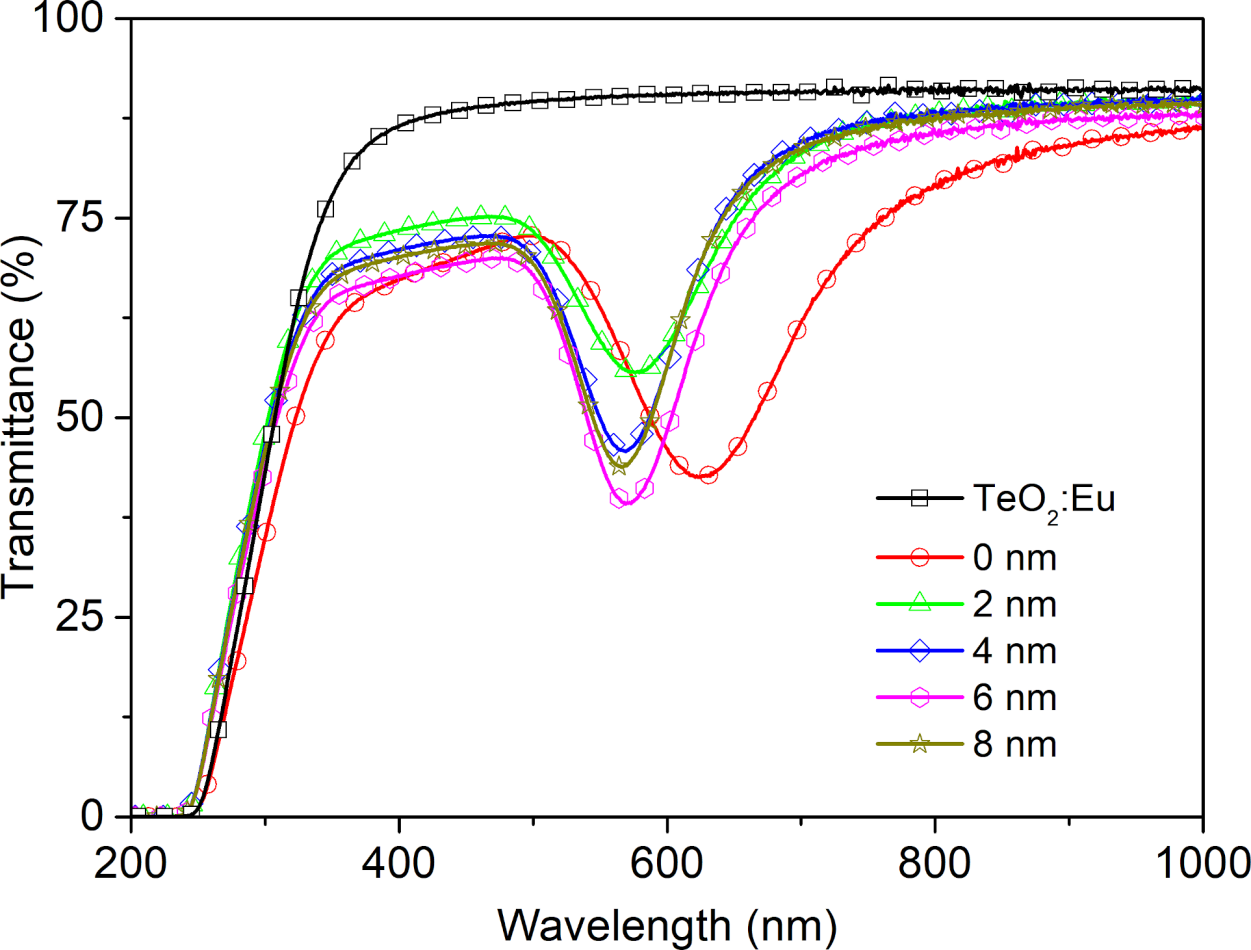
UV–vis spectra of a TeO_2_:Eu film and TeO_2_:Eu films deposited on the plasmonic platform without a dielectric layer (0 nm) and with a dielectric Al_2_O_3_ layer with different thickness, in a range of 2 to 8 nm.

**Figure 10:**
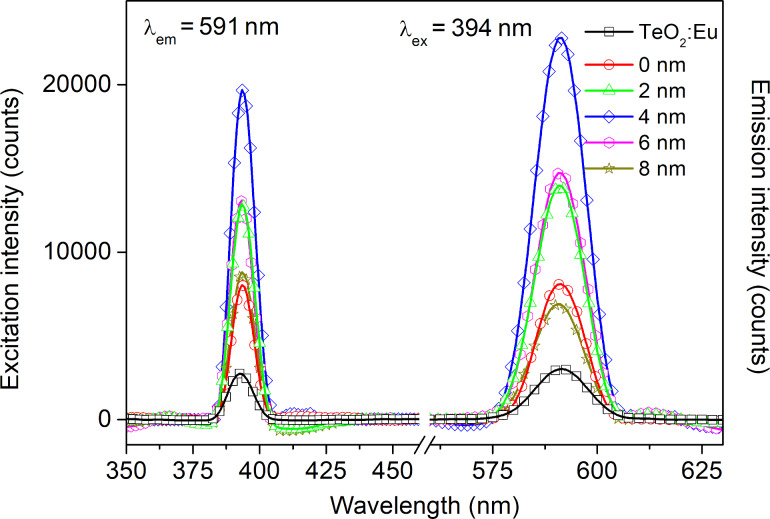
Excitation and emission spectra of TeO:Eu-based structures.

**Figure 11:**
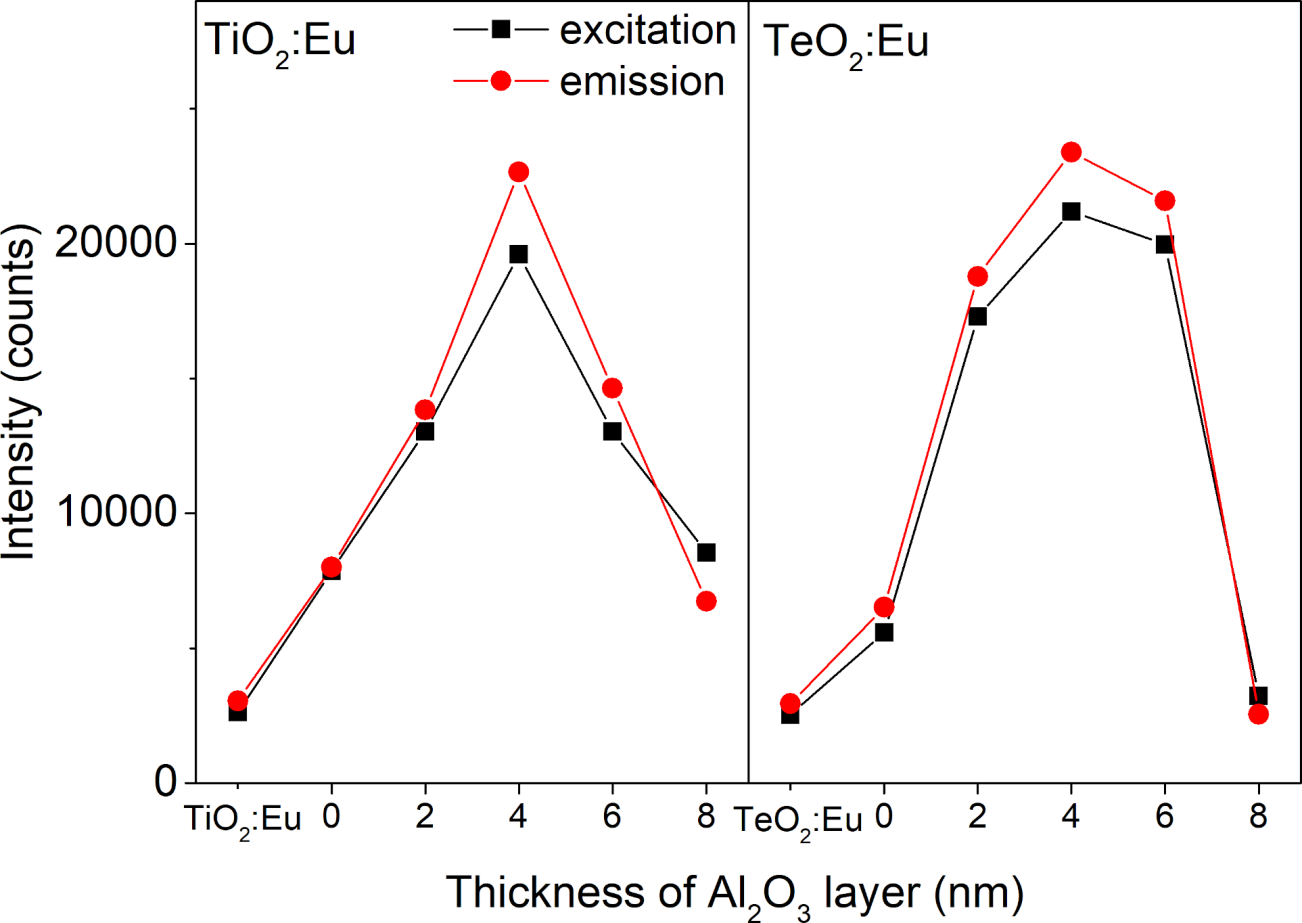
Comparision of the maxmimum of ecxitation and emission peaks for TiO_2_:Eu and TeO_2_:Eu thin films, and for films deposited on the plasmonic platform without a dielectric layer (0 nm) and with a dielectric Al_2_O_3_ layer with different thickness, in a range of 2 to 8 nm.

To compare optical properties and investigate the influence of plasmonic resonance on the intensity of luminescence, transmission and luminescence spectra were recorded for the single luminescent oxide layer and the luminescent films deposited on gold nanostructures, in both configurations with and without dielectric Al_2_O_3_ layer. The transmittance spectra in a range of 200–1000 nm are presented in Figure 9. The TeO_2_:Eu layer is characterized by a high transmission intensity with a transmittance of approximately 85% in the visible range. Multilayers with gold nanostructures exhibit a minimum, which is ascribed to plasmonic resonance. The shift of the resonance wavelength in a multilayer, as well as the change of transmission band intensity can be explained by the change of plasmonic platform surrounding. In comparison with air both TeO_2_:Eu and Al_2_O_3_ layers exhibit a much greater permittivity, which affects the optical properties of plasmonic nanostructures and redshifts the resonance wavelength [34–35,40–41]. The excitation and emission spectra samples are shown in Figure 10. One main spectral line can be distinguished on excitation spectra for all samples. It is positioned at 394 nm and corresponds to the ^7^F_0_→^5^L_6_ transition in Eu^3+^. As far as absorption and excitation of Eu^3+^ ions are concerned, it is the most probable and most frequently observed transition according to literature [23,42–43]. Samples deposited on Au plasmonic platforms show an increased intensity of the excitation peak. Moreover, samples with an additional aluminium oxide ultrathin film exhibit a higher excitation intensity. The increased excitation for samples with Au nanostructures may be ascribed to the plasmonic resonance effect, which results in greater absorption of light and, consequently, more efficient excitation of the luminescent material. The changing of the surrounding of plasmonic structures has visible impact on the luminescent properties of a sample. The redshift of the resonance wavelength and the decrease of transmittance observed in Figure 9 caused by Al_2_O_3_ may successfully tune the optical properties of gold plasmonic nanostructures to obtain a more efficient excitation of the luminescent layer with europium ions. The characteristic narrow emission band at 591 nm was assigned to the ^5^D_0_→^7^F_1_ magnetic dipole transition in Eu^3+^ [40–41]. Its intensity is higher for samples with Au platforms than for the reference sample (TeO_2_:Eu) and reaches the greatest value for 4 nm of Al_2_O_3_ layer. In general, greater excitation of luminescent material leads to more efficient emission. The observed magnetic dipole transition is characteristic for a uniform distribution of luminescent ions in a host matrix. Usually, such centrosymmetric matrices are crystalline structures or consist of some crystal areas. The intensity of excitation and emission peaks for titanium dioxide- and tellurium dioxide-based structures, are presented in Figure 11. The luminescence intensity and the influence of the plasmonic platform for both kinds of phosphors is comparable.

**Figure 12:**
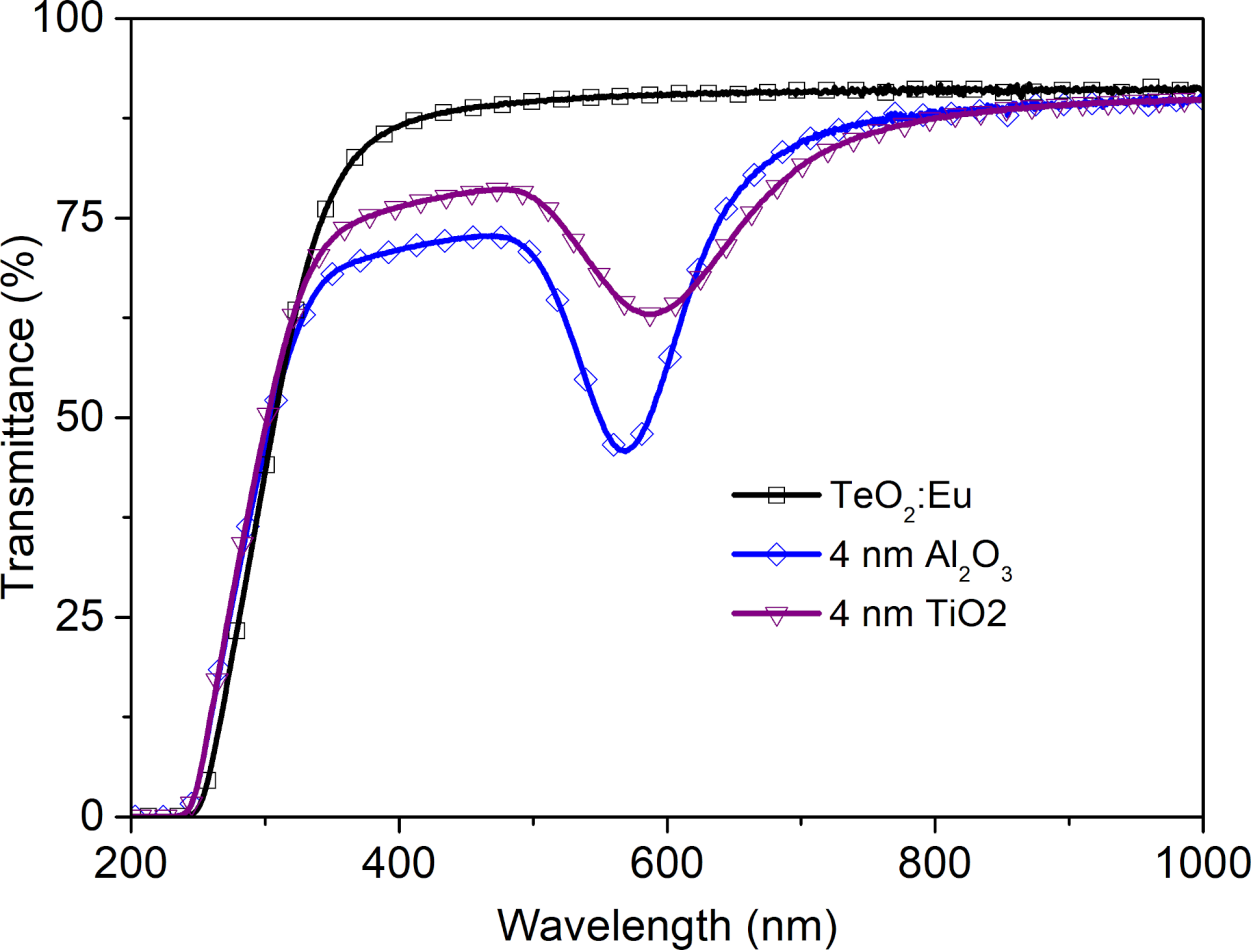
UV–vis spectra of a TeO_2_:Eu film and TeO_2_:Eu films deposited on a plasmonic platform with dielectric Al_2_O_3_ or TiO_2_ layer with a thickness of 4 nm.

**Figure 13:**
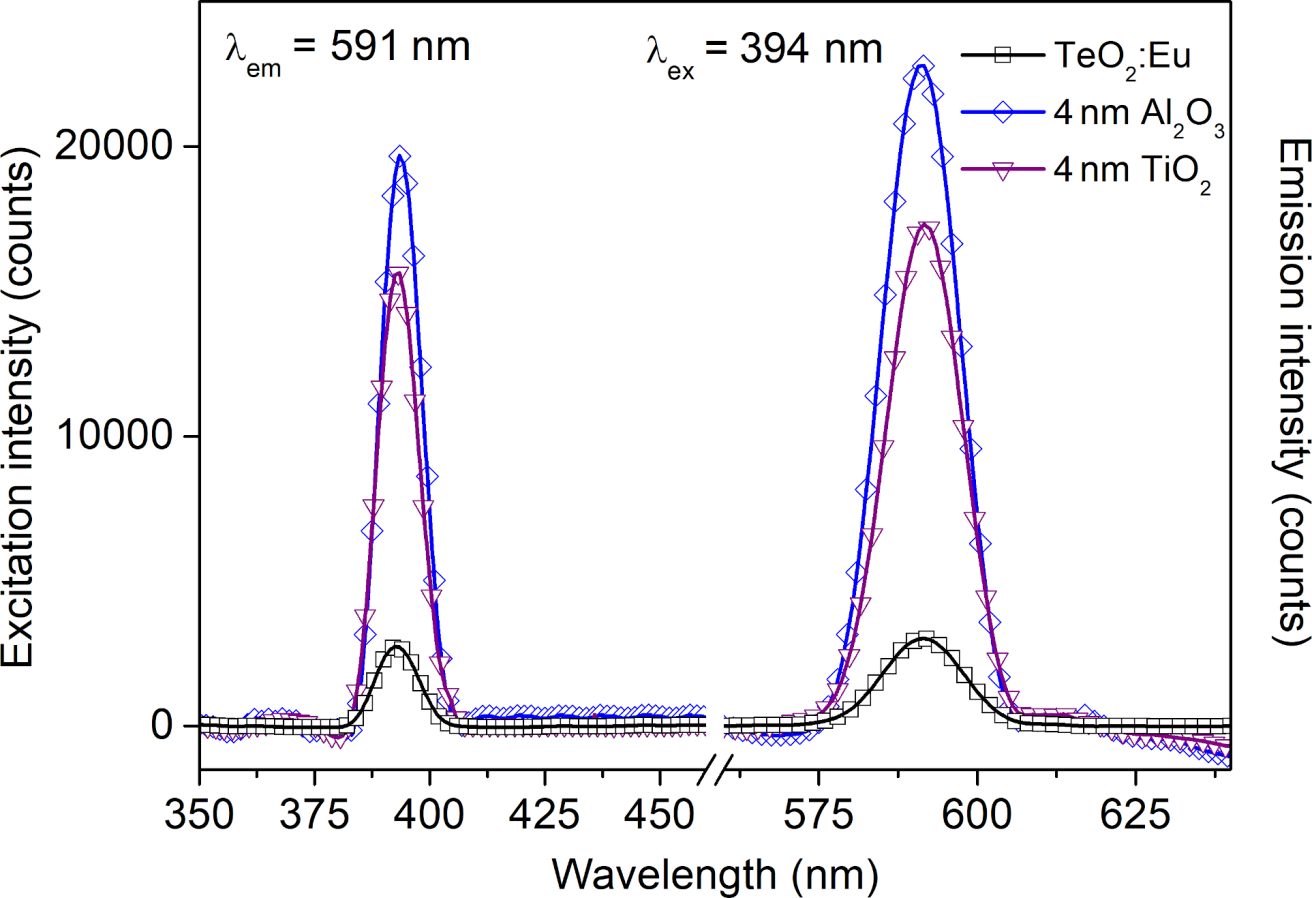
Excitation and emission spectra of a TeO_2_:Eu film and TeO_2_:Eu films deposited on a plasmonic platform with dielectric Al_2_O_3_ or TiO_2_ layer with a thickness of 4 nm.

Additionally, we decided to change the dielectric layer from Al_2_O_3_ to TiO_2_. It enabled the production of structures in a one-step process during the deposition of multilayers by magnetron sputtering. On the basis of emission spectra for structures with alumina oxide, 4 nm of titanium dioxide was selected as a dielectric layer. The optical characteristics are presented in Figure 12 and Figure 13. Because of the different dielectric constants of both types of dielectric layers, a shift of plasmon resonance position is observed (Figure 12). From the luminescence spectra in Figure 13 it can be seen that it is possible to enhance of luminescence intensity by an ultrathin TiO_2_ film, almost as much as by Al_2_O_3_.

## Conclusion

In this work we showed an interesting material in which europium ions dispersed in a thin TiO_2_ or TeO_2_ film are responsible for the emission of light. The intensity of the luminescence can be enhanced by the plasmon resonance from Au nanostructures. The gain is tunable by the thickness of a Al_2_O_3_ thin film deposited as a separator between metallic nanostructures and the luminescent layer. Interestingly, no transition caused by the electric dipole (615 nm, typical for Eu^3+^ ions) was observed in the emission spectrum for both kinds of metal oxide matrix. The main emission peak corresponds to the transition occurring via magnetic dipole, which is independent of the host matrix. Such a phenomenon usually takes place in materials in which Eu^3+^ ions are located at sites with higher symmetry, and explaining this behavior in the presented samples would require further research. Anyway, we believe that the presented structures could be attractive for optoelectronic applications.
